# An interpretable model based on graph learning for diagnosis of Parkinson’s disease with voice-related EEG

**DOI:** 10.1038/s41746-023-00983-9

**Published:** 2024-01-05

**Authors:** Shuzhi Zhao, Guangyan Dai, Jingting Li, Xiaoxia Zhu, Xiyan Huang, Yongxue Li, Mingdan Tan, Lan Wang, Peng Fang, Xi Chen, Nan Yan, Hanjun Liu

**Affiliations:** 1 Department of Rehabilitation Medicine, The First Affiliated Hospital, Sun Yat-sen University, Guangzhou, China; 2 School of Biomedical Engineering, Sun Yat-sen University, Shenzhen, China; 3 CAS Key Laboratory of Human-Machine Intelligence-Synergy Systems, Shenzhen Institute of Advanced Technology, Chinese Academy of Sciences, Shenzhen, China; 4 Department of Rehabilitation Medicine, Zhujiang Hospital, Southern Medical University, Guangzhou, China; 5 Guangdong-Hong Kong-Macao Joint Laboratory of Human-Machine Intelligence-Synergy Systems, Shenzhen Institute of Advanced Technology, Chinese Academy of Sciences, Shenzhen, China; 6 Guangdong Provincial Key Laboratory of Brain Function and Disease, Zhongshan School of Medicine, Sun Yat-sen University, Guangzhou, China

**Keywords:** Diagnostic markers, Diagnostic markers

## Abstract

Parkinson’s disease (PD) exhibits significant clinical heterogeneity, presenting challenges in the identification of reliable electroencephalogram (EEG) biomarkers. Machine learning techniques have been integrated with resting-state EEG for PD diagnosis, but their practicality is constrained by the interpretable features and the stochastic nature of resting-state EEG. The present study proposes a novel and interpretable deep learning model, graph signal processing-graph convolutional networks (GSP-GCNs), using event-related EEG data obtained from a specific task involving vocal pitch regulation for PD diagnosis. By incorporating both local and global information from single-hop and multi-hop networks, our proposed GSP-GCNs models achieved an averaged classification accuracy of 90.2%, exhibiting a significant improvement of 9.5% over other deep learning models. Moreover, the interpretability analysis revealed discriminative distributions of large-scale EEG networks and topographic map of microstate MS5 learned by our models, primarily located in the left ventral premotor cortex, superior temporal gyrus, and Broca’s area that are implicated in PD-related speech disorders, reflecting our GSP-GCN models’ ability to provide interpretable insights identifying distinctive EEG biomarkers from large-scale networks. These findings demonstrate the potential of interpretable deep learning models coupled with voice-related EEG signals for distinguishing PD patients from healthy controls with accuracy and elucidating the underlying neurobiological mechanisms.

## Introduction

Parkinson’s disease (PD) is a neurodegenerative disorder that exerts a profound impact on the quality of life for 7–10 million people worldwide^1,2^. It is characterized by progressive and diverse symptoms that involve both motor and non-motor impairments. However, the pathogenic mechanism of PD remains poorly understood, with only 20% of cases being attributed to specific genetic factors^3,4^. Therefore, the precise and early diagnosis of PD continues to present considerable challenges, as this holds significance for effective clinical management.

One promising avenue for PD diagnosis lies in the identification of reliable biomarkers across various behavior domains, including handwriting patterns^5^, motor function^6^, gait patterns^7^, and speech characteristics^8^. Of particular interest, resting-state electroencephalography (EEG) has emerged as a potential diagnostic tool for PD diagnosis due to its noninvasiveness, cost-effectiveness, and ability to capture brain activity with high-temporal resolution^9–11^. Quantitative EEG (QEEG) measures, including power spectral density^10,11^ and spatiotemporal microstates^9^, have been extracted as distinctive features to distinguish PD patients from healthy individuals. More recently, an increasing number of studies on PD diagnosis have shifted towards integrating deep learning techniques with large-scale EEG networks^12,13^. For example, Oh et al. ^14^ utilized a thirteen-layer convolutional neural network (CNN) for identifying resting-state EEG data from PD patients, achieving a remarkable classification accuracy (ACC) of 88.25%. Chaturvedi et al. ^15^ integrated resting-state EEG parameters with least absolute shrinkage and selection operator (LASSO) and achieved an area under the curve (AUC) of 0.76 in PD diagnosis. These methods, however, rely heavily on their assumption of stationarity and integrability of the EEG signals^9^, which may not be valid given the dynamic nature of PD-related changes in brain activity. Therefore, the need to capture the stable and time-varying patterns, which cannot be adequately addressed by the stochastic resting-state EEG signals, arises as a challenge to extract discriminative features for distinguishing PD patients from healthy individuals.

In contrast to resting-state EEG signals, task-related EEG signals exhibit phase- and time-locked responses to motor and non-motor tasks and their functional networks/connectivity. This aspect offers valuable insights into PD-related alterations in neural activity and extraction of distinct EEG features between PD patients and healthy controls. A particular area of interest lies in motor speech disorders, which affect approximately 90% of PD patients and are considered as one of the premotor symptoms^16,17^. Previous studies have demonstrated that PD patients are impaired in sensorimotor control of vocal production^18–22^, which is manifested as enhanced event-related potential (ERP) P2 responses to pitch perturbations in voice auditory feedback^23–25^. This observation suggests the potential of machine learning-based extraction of salient features from voice-related EEG signals to obtain robust biomarkers for PD diagnosis. Notably, Shi et al. ^26^ developed four deep learning architectures, namely the CNN, the Recurrent Neural Network (RNN) and two hybrid models (2D-CNN-RNN and 3D-CNN-RNN), for PD diagnosis based on voice-related EEG signals. Their findings showed that the hybrid models outperformed the conventional models, achieving accuracies of 82.89% (3D-CNN-RNN), 81.13% (2D-CNN-RNN), 80.89% (CNN), and 76.00% (RNN). This study provides preliminary evidence for the feasibility of deep learning models with task-related EEG signals to distinguish PD patients from healthy individuals.

Nevertheless, the complexity of deep learning models has raised concerns regarding their interpretability, particularly in understanding what features they can learn from EEG signals and how they relate to the clinical characteristics of PD patients. Therefore, there is a growing demand for enhancing the interpretability of deep learning methods^27–29^. One promising interpretable model is the multi-category mutual verification^30^, which integrates graph learning and neurophysiological information models. The graph convolutional network (GCN), a key component of the graph learning model, has achieved strong performance in diagnosing neurodegenerative diseases using neuroimaging data^31^. The neurophysiological information model involves large-scale neural networks for EEG microstate analysis, which has been successfully used for PD diagnosis with resting-state data^9^. However, no studies have yet combined these two methods to enhance model interpretability in PD diagnosis using task-related EEG signals.

**Fig. 1:**
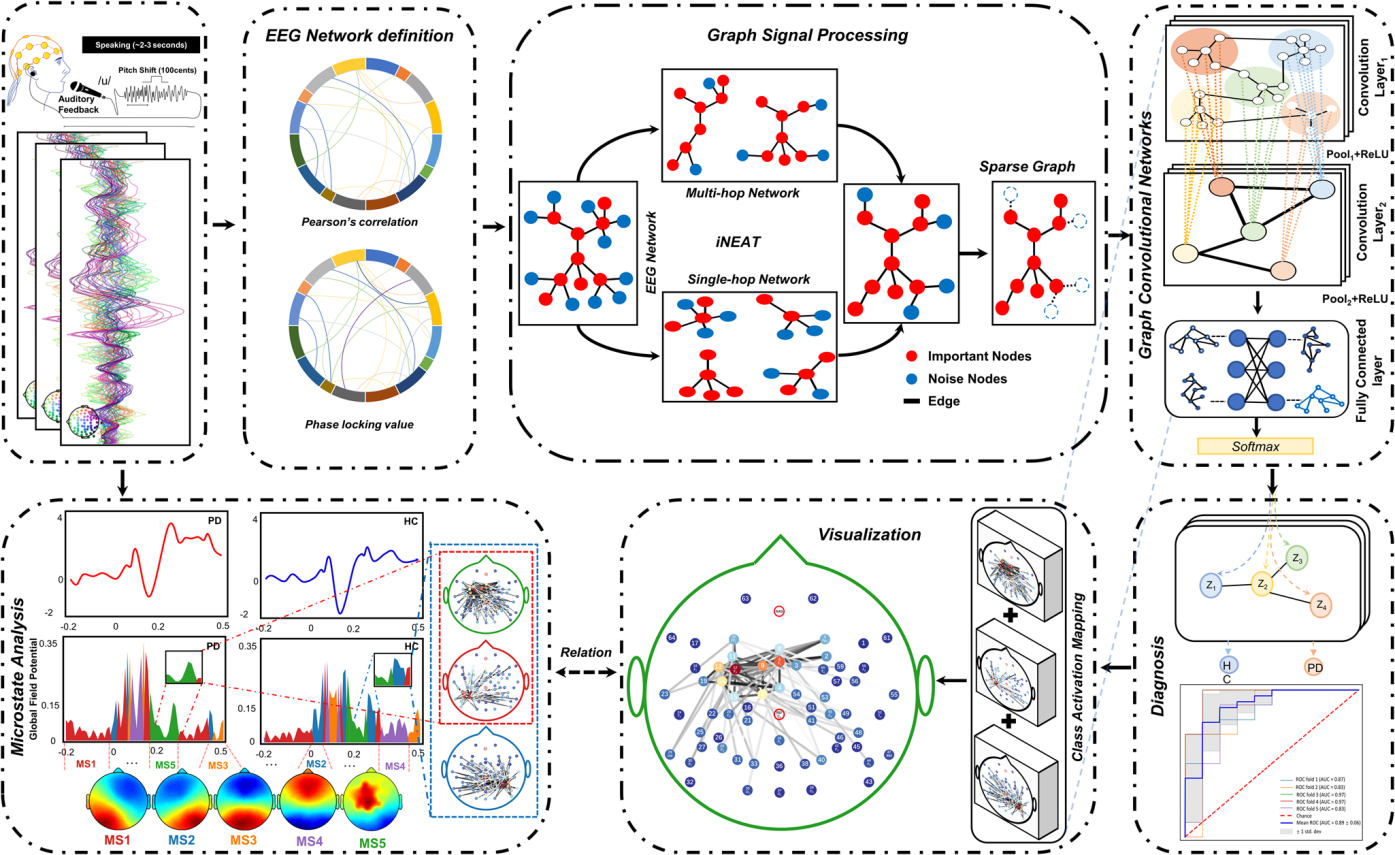
The schematic diagram of the GSP-GCNs model designed for PD diagnosis. The GSP module is responsible for acquiring collaborative graph information of both single-hop and multi-hop networks from the EEG signals, while the GCNs module automatically learns the graph structure by aggregating the information of neighboring nodes. INA incomplete network alignment, GCN graph convolutional network, GSP graph signal processing.

To this end, the present study proposes a novel knowledge-guided framework (see Fig. 1), graph signal processing-graph convolutional networks (GSP-GCNs), to distinguish PD patients from healthy individuals using large-scale EEG network obtained from a specific task involving the manipulation of auditory feedback during vocal production. This framework consists of four sequential components: GSP, the graph-network module, the classifier, and the interpretable model. Firstly, the GSP module analyzes and processes the large-scale EEG networks to identify dynamic connectivity patterns. Subsequently, the graph-network module captures these connectivity patterns as key features for classification. The classifier component then utilizes these extracted features to discriminate PD patients from healthy individuals. Lastly, the interpretable model is incorporated to enhance the interpretability of the framework by providing a global visualization of essential learned features of the model and aligning them with voice-related EEG microstates characteristics. By adopting this innovative approach, our GSP-GCNs framework aims to provide illustrative information for facilitating the use of the deep learning model in PD diagnosis with task-related EEG data.

## Results

### Comparison of classified performance

The present study proposed four graph-network-based models: PCC+GCNs, PLV+GCNs, PCC + GSP-GCNs, and PLV + GSP-GCNs. The PCC + GSP-GCNs model constructs the brain network using PCC features and employs the GSP-GCNs model as the classifier. The PLV + GSP-GCNs model is similar to the PCC + GSP-GCNs model, except that it uses PLV features to construct brain networks. In contrast, the PCC+GCNs and PLV+GCNs models do not include the GSP module based on the aforementioned methods.

**Fig. 2:**
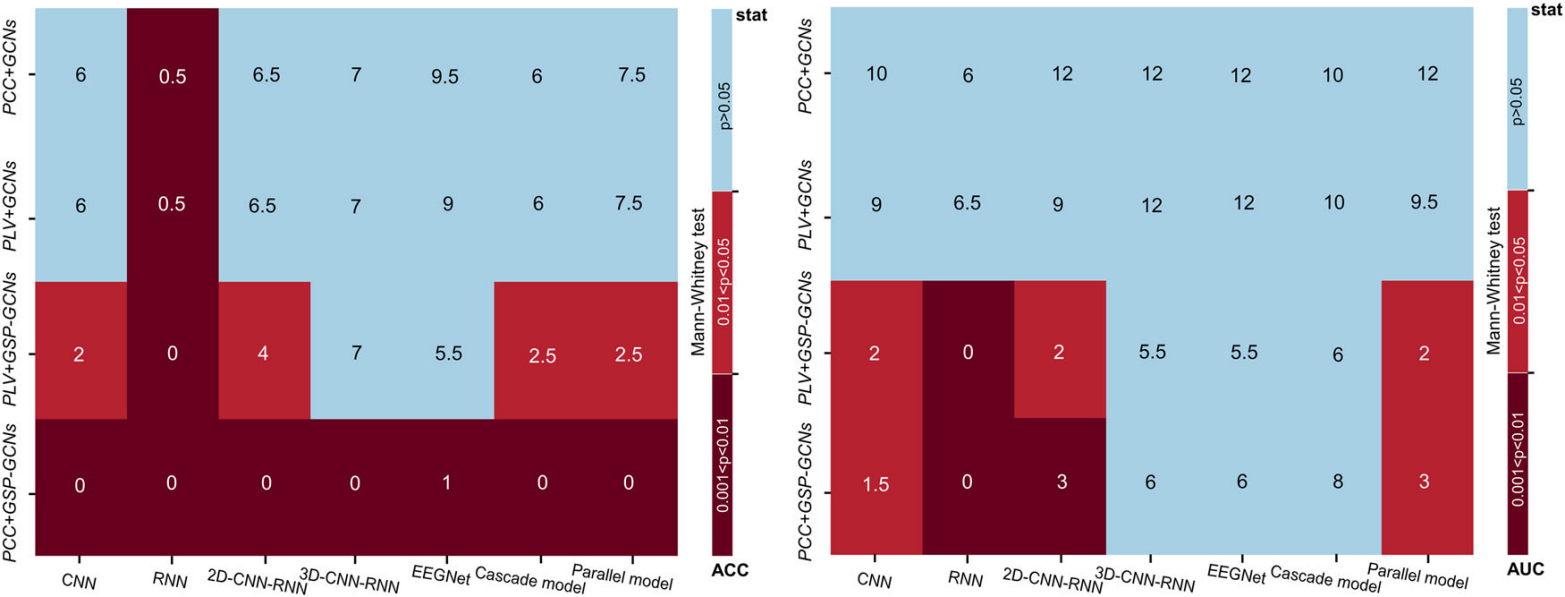
Heatmaps showing statistical differences in classification performance between models. The left and right panels show the resutls of non-parametric Mann-Whitney tests comparing the GCNs model and conventional deep learning models in 5-fold cross validation for classification performance corresponding to ACC and AUC, respectively. Each cell in the heatmap represents the statistical significance of differences between the models, with light blue indicating no significance, red indicating a significant difference, and dark red denoting a higher level of significance.

Table 1 provides the classification performance achieved by our proposed models and baseline models based on voice-related EEG signals, and Fig. 2 shows the results of non-parametric Mann-Whitney tests with Bonferroni correction that compare the classification performance (ACC and AUC) of these models in 5-fold cross validation. Our PCC + GSP-GCNs model outperformed the EEGNet model significantly, with an accuracy increase of 7.9% (82.3% vs. 90.2%), an AUC increase of 7.9% (81.2% vs. 89.1%), a sensitivity boost of 2.5% (81.5% vs. 84.0%), and a specificity enhancement of 8.8% (79.6% vs. 88.4%). As well, our proposed graph-based models exhibit superior performance compared to previous methods that did not use graph learning^26^. Specifically, the PLV + GSP-GCNs model improved those metrics by 8.2%, 7.7%, 3.6%, and 10.2%, compared to the 3D-CNN-RNN model. In addition, Table 1 shows the computational complexity of our proposed models and baseline models. The computational complexity of the GCNs model is 2*O(*nlogn*)+O(*n*^*2*^)^32,33^ while the computational complexity of the baseline models ranges from O(*n*·*d*^2^)+O(*n*^2^) to 3*O(*k*·*n*·*d*^2^)+2*O(*n*·*d*^2^)+2*O(*n*^2^)^26,34–36^, where k denotes the convolution kernel size (greater than 3), d denotes the time series length (d = 700), and n denotes the number of channels (*n* = 64). Accordingly, our GCNs model shows a considerably lower computational complexity than all baseline models.

**Table 1:** Classification performance and computational complexity of different deep learning models based on voice-related EEG signals, where *k* represents the convolution kernel size, *d* represents the time series length, and *n* represents the number of channels.

Models		Evaluation Criterion				
		*ACC*	*AUC*	*Sensitivity*	*1-Specificity*	*Computational Complexity*
*Baseline Models*	CNN	79.6%	78.1%	80.2%	77.5%	4*O(*k*·*n*·*d*^2^)+O(*n*^2^)
	RNN	75.2%	74.2%	80.1%	75.3%	O(*n*·*d*^2^)+O(*n*^2^)
	2D-CNN-RNN	81.6%	79.5%	78.6%	80.2%	O(*k*·*n*·*d*^2^)+O(*n*·*d*^2^)+O(*n*^2^)
	3D-CNN-RNN	82.1%	81.2%	83.1%	78.2%	2*O(*k*·*n*·*d*^2^)+O(*n*·*d*^2^)+O(*n*^2^)
	EEGNet	82.3%	81.2%	81.5%	79.6%	3*O(*k*·*n*·*d*^2^)+O(*n*^2^)
	Cascade model	80.4%	83.2%	82.1%	77.2%	3*O(*k*·*n*·*d*^2^)+2*O(*n*·*d*^2^)+2*O(*n*^2^)
	Parallel model	81.1%	80.2%	82.3%	79.8%	3*O(*k*·*n*·*d*^2^) + O(*n*^2^)
*GCNs*	PCC+GCNs	84.1%	83.1%	78.2%	86.0%	2*O(*nlogn*)+O(*n*^2^)
	PLV+GCNs	84.8%	82.2%	79.1%	84.3%	2*O(*nlogn*)+O(*n*^2^)
	PLV + GSP-GCNs	88.9%	87.5%	86.1%	86.2%	2*O(*nlogn*)+O(*n*^2^)
	PCC + GSP-GCNs	90.2%	89.1%	84.0%	88.4%	2*O(*nlogn*)+O(*n*^2^)

*ACC* accuracy, *AUC* area under curve, *CNN* convolutional neural network, *RNN* recurrent neural networks, *GSP* graph signal processing, *GCN* graph convolutional network, *PCC* Pearson correlation coefficient, *PLV* phase locking value.

**Fig. 3:**
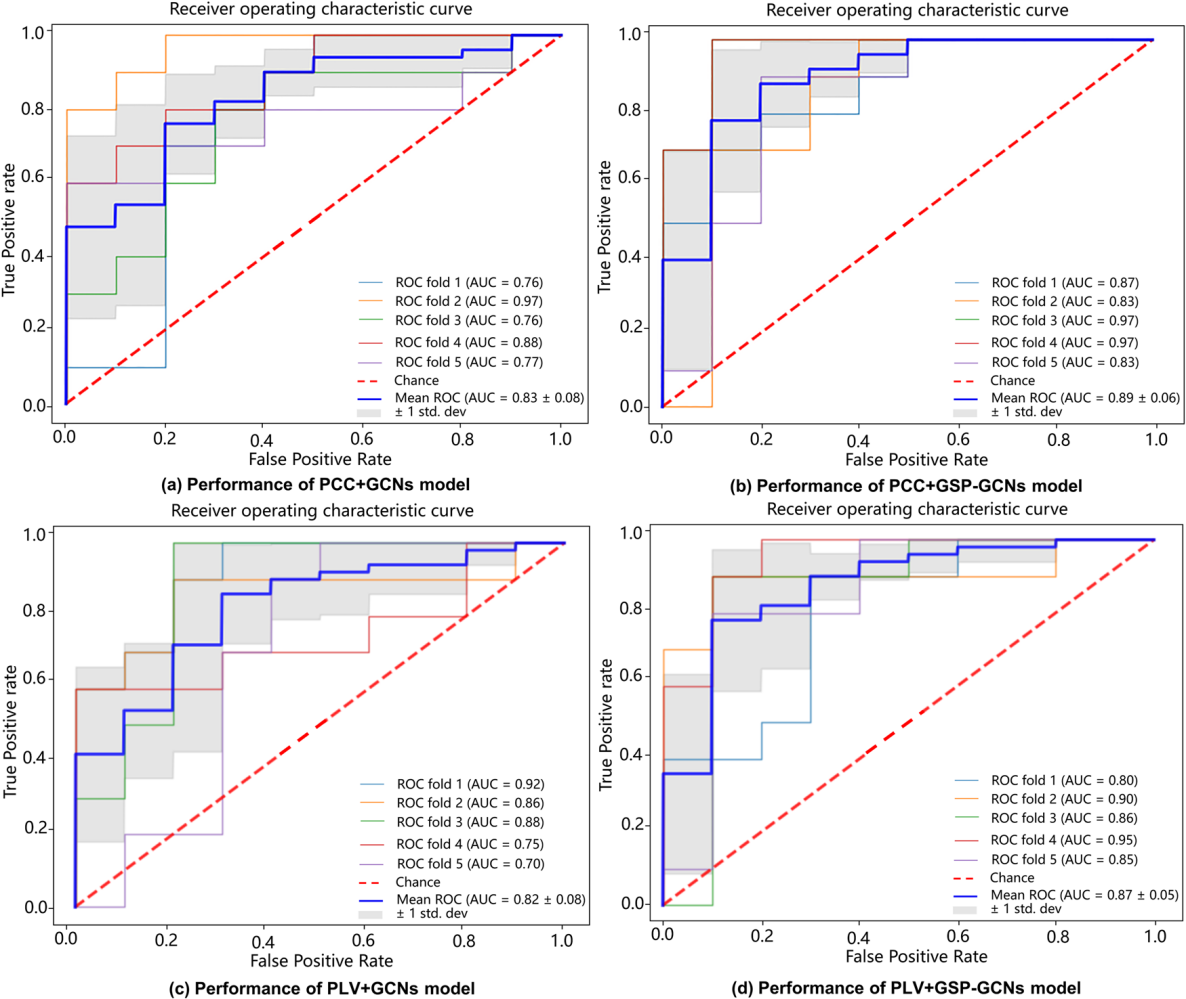
The ROC curves of the GCNs models. Panels **a**–**d** show the ROC curves for the PCC+GCNs, PLV+GCNs, PCC + GSP-GCNs, and PLV + GSP-GCNs models, respectively. The solid blue line represents the average performance across different test sets using 5-fold cross-validation, while the other colored lines show the performance of individual test set. The gray shaded area indicates the variance range of the average ROC curve.

Notably, Table 1 and Fig. 2 also show a comparison of classification performance across our proposed models. The GSP-GCNs models exhibited significantly superior performance compared to the GCNs models, regardless of the feature type. For example, the PCC + GSP-GCNs model achieved higher ACC, AUC, sensitivity, and specificity than the PCC+GCNs model by 6.1% (84.1% vs. 90.2%), 6.0% (83.1% vs. 89.1%), 5.8% (78.2% vs. 84%), and 2.4% (86.0% vs. 88.4%), respectively. Similarly, the PLV + GSP-GCNs model improved these metrics by 4.1% (84.8% vs. 88.9%), 5.3% (82.2% vs. 87.5%), 7.0% (79.l% vs. 86.l%), and 1.9% (84.3% vs. 86.2%) compared to the PLV+GCNs model. Furthermore, we employed a cross-validation approach to verify the out-of-distribution detection capability and stability of the GCNs model. Figure 3 indicates a consistently low variance of receiver operating characteristic (ROC) values, all remaining below 0.08 for our proposed models. These results highlight the potential of GSP processing in enhancing the classification performance by balancing the local and global information of single-hop and multi-hop networks through graph aggregation.

### Interpretable GSP-GCN model

**Fig. 4:**
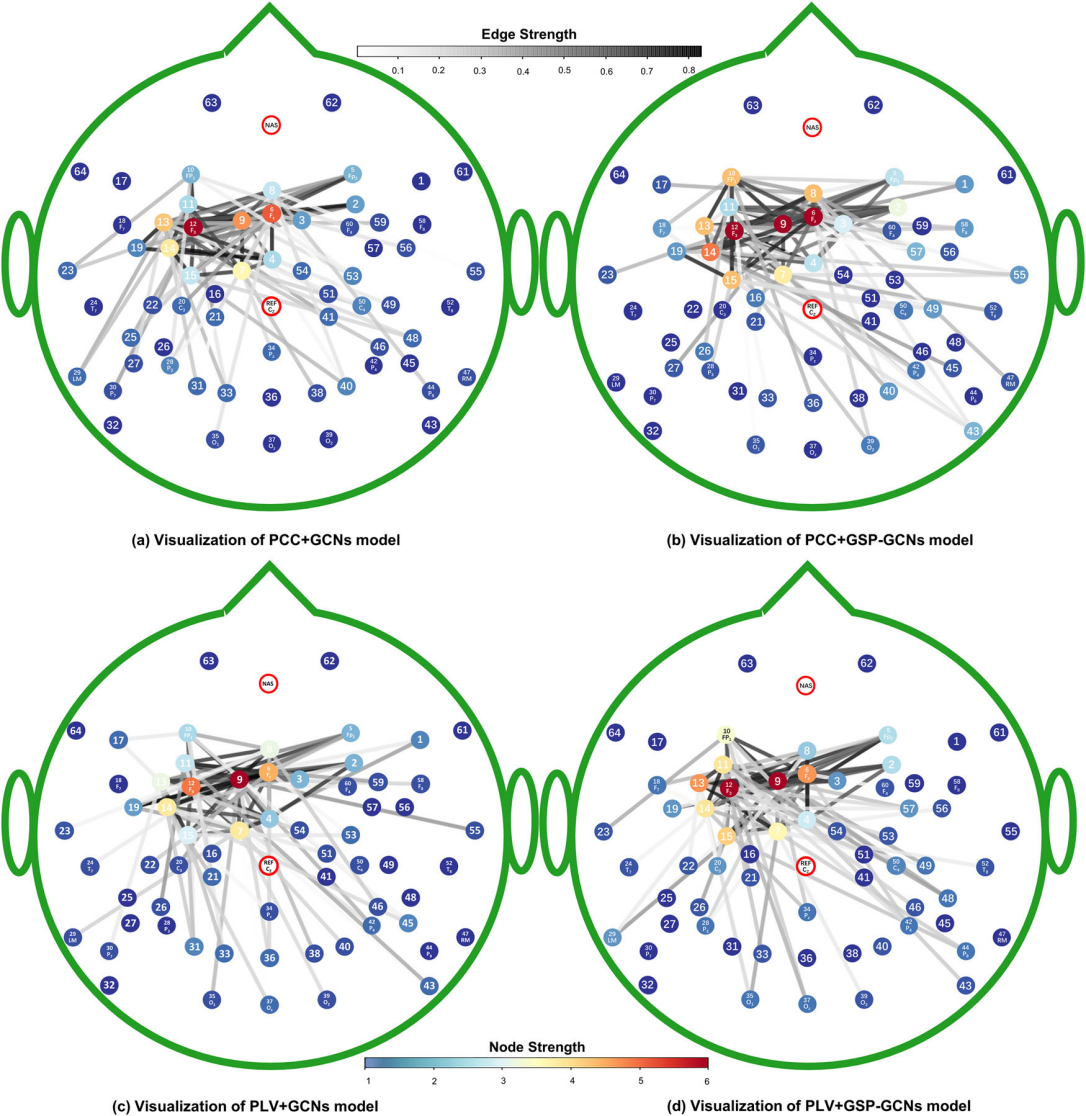
The saliency maps of the discriminative biomarkers learned by the GCNs models for the FAF task. The numbers ranging from 1 to 64 correspond to the electrodes according to the international EEG 10-20 system. Panels **a**–**d** show the distribution of the contribution network for the PCC+GCNs model, the PCC + GSP-GCNs model, the PLV+GCNs model, and the PLV + GSP-GCNs model, respectively. Nodes with a redder hue indicate greater contributions to the model, and edges with a blacker hue indicate higher contributions.

The interpretability of the GSP-GCNs framework was assessed in the present study, aiming to capture global frequency-spatial-temporal dependencies in the large-scale EEG network and extract essential information for decoding tasks from the time series data. We used a modified CAM method to visualize the global representation learned by our models and generate their saliency maps in the context of vocal pitch regulation. Figure 4 illustrates the discriminative distributions of large-scale EEG networks obtained from the FAF task and learned by the PCC+GCNs and the PCC + GSP-GCNs models. The most significant discriminative distributions were located in the left ventral premotor cortex (vPMC), superior temporal gyrus (STG), and Broca’s area. These results suggest that the GSP-GCNs models have the capability to capture the intrinsic representation of the major brain activity difference during vocal motor control between PD patients and healthy controls.

**Fig. 5:**
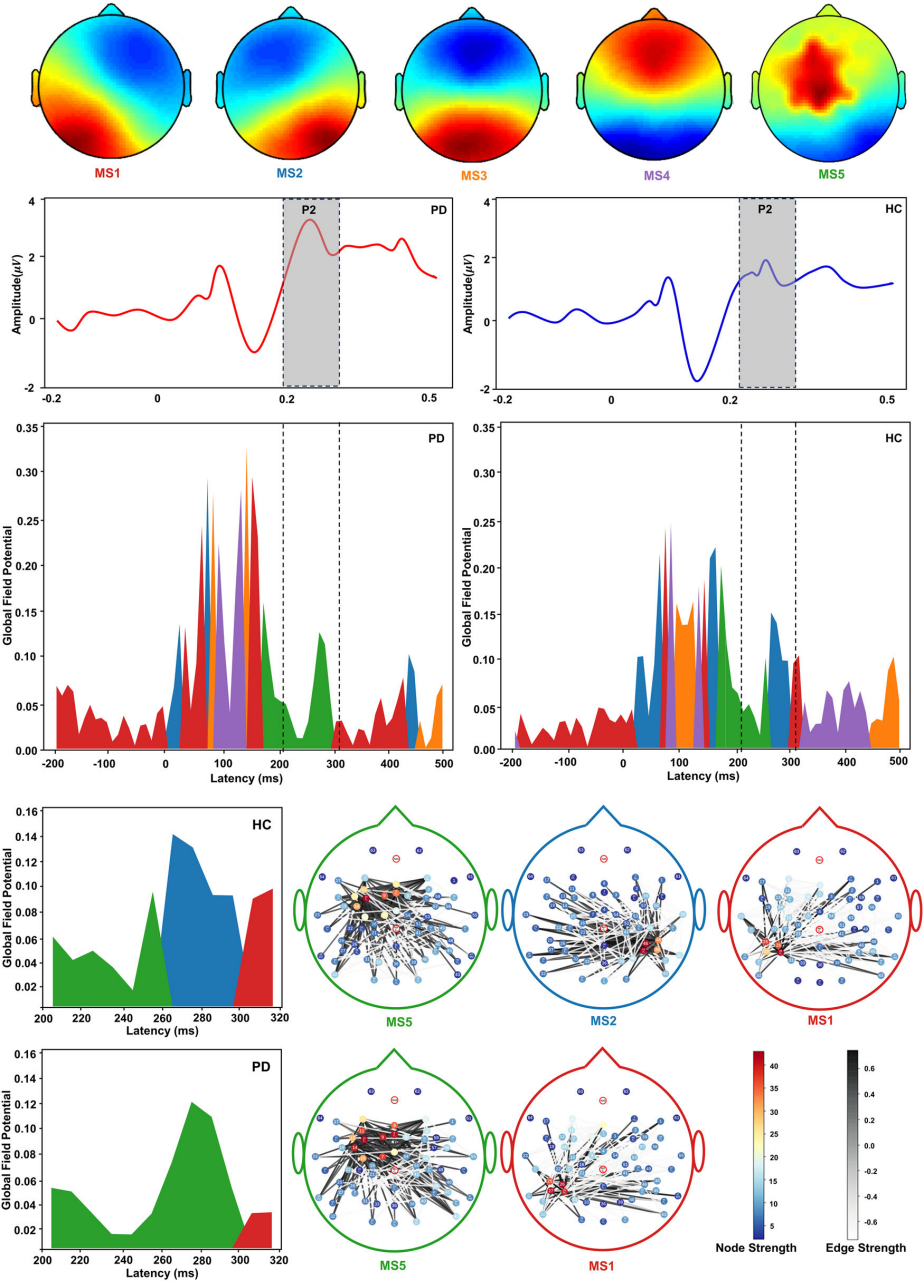
The distribution of microstate classes corresponding to grand-averaged ERP in the FAF task for PD and HC groups. Different colors represent different microstates. Five microstates clustered by k-means algorithm are shown in the first row, average time potential fluctuations with emphasis on P2 component are shown in the second row, global field power is shown in the third row, and functional activity distributions within microstates and EEG networks associated with P2 component are shown in the bottom. GFP global field power, MS microstate.

Moreover, we performed microstate analysis on the voice-related EEG signals from PD patients and healthy controls and identified five distinct microstates (MS1-MS5) with high energy fluctuation in the time frame of 0–300 ms (see Fig. 5). The most prominent microstate was the MS5, located in the electrodes near the left vPMC and Broca’s area in the time frame of 205–315 ms. This temporal correspondence aligns with the large-scale EEG networks associated with the P2 component. The microstate transitioned from MS5 to MS2 during the 260–300 ms period of P2, with MS2 located near the right STG. The network identified in the MS5 microstate resembles the discriminative distributions learned by the GSP-GCNs model, further strengthening the interpretability of our proposed models.

## Discussion

The present study proposed a novel, interpretable deep learning framework based on GSP-GCNs to distinguish from PD patients from healthy controls using voice-related EEG signals. By incorporating both local and global information from single-hop and multi-hop networks, our GSP-GCNs model achieved an averaged classification accuracy of 90.2% and exhibited a performance improvement of 9.5% compared to other deep learning models. Moreover, the interpretability analysis of our GSP-GCNs models revealed discriminative distributions of large-scale EEG networks and topographic map of the microstate MS5 in the P2 time window, primarily located in the left vPMC, STG, and Broca’s area that have been implicated in PD-related motor speech disorders. Overall, our proposed GSP-GCNs models offer a valuable tool for PD diagnosis based on the interpretable results derived from large-scale voice-related EEG networks.

A growing body of studies has concentrated on the development of PD diagnosis by integrating resting-state EEG with machine learning techniques^12–14,37–39^. Recently, several deep learning models have been proposed for PD diagnosis using different resting-state EEG datasets (e.g. the public UNM dataset, public SanDiego dataset). For example, Oh et al. ^14^ proposed a thirteen-layer CNN model while Lee et al. ^39^ proposed a hybrid CNN-RNN model, achieving classification accuracies of 88.25% and 99.2% in distinguishing PD patients form healthy controls, respectively. In addition, Shah et al. ^38^ developed a dynamical system generated hybrid network (DGHNet) for successful categorization of on-medication vs. off-medication PD patients with a classification accuracy of 99.2%. Nevertheless, the practicality of these models is constrained by the prolonged training period, the large datasets requirement, and the lack of interpretability of specific EEG features in PD diagnosis. Furthermore, the stochastic nature of resting-state EEG poses challenges in capturing the distinct patterns of PD-related brain activity.

In attempts to address these limitations, Shi et al. ^26^ developed four deep learning architectures (i.e. CNN, RNN, 2D-CNN-RNN, 3D-CNN-RNN) using voice-related EEG signals during vocal pitch regulation for PD diagnosis and achieved classification accuracies ranging from 76.00% to 82.89%. Hassin-Baer et al. ^40^ applied machine learning models for the diagnosis of early-stage PD using the event-related EEG signals during visual Go/No-Go and auditory Oddball cognitive tasks, achieving an AUC of 0.79 and identifying a total of 15 EEG features. In the present study, our proposed the GSP-GCNs models used voice-related EEG signals similar to Shi et al. ^26^ and achieved classification accuracies ranging from 84.1% to 90.2%, indicating a remarkable performance superiority over those models proposed by Shi et al. ^26^. Furthermore, our PCC + GSP-GCNs model also outperformed other models (see Table 1 and Fig. 2), including the EEGNet model, that do not use graph learning. Taken together, these findings demonstrate the effectiveness of deep learning models with graph learning and voice-related EEG signals for PD diagnosis.

Our proposed GSP-GCNs models, integrated with voice-related EEG, present a significant advancement over other deep learning models for PD diagnosis in several aspects. A significant strength lies in their capacity to combine the global and local properties of brain functional networks using single-hop and higher-order networks, enabling the aggregation of information from the nodes of the functional brain networks and the extraction of features for graph learning. Another strength is the use of GCN models for graph data, which have advantages over other convolutional neural networks, such as capturing complex node relationships, adapting to different graph properties, and handling large-scale network data. In contrast, the CNN-based approaches only focus on local features due to their limited perceptual field but ignore the global correlations among EEG signals. Similarly, the CNN-RNN approaches only capture the sequential relationships of EEG signals while overlooking the relevance of the EEG connectivity network. On the other hand, resting-state EEG signals are stochastic in nature and fail to capture the functional specificity of brain activity related to PD when compared to task-related EEG signals. The present study used a task that involves auditory-motor integration for vocal pitch regulation, which reveals the neural processes involved in the coordination of sensory and motor systems for speech production. These processes have been demonstrated to be impaired in PD^21–25^. Therefore, our GSP-GCNs models provide a novel way for PD diagnosis using voice-related EEG signals and deep learning techniques, which is more reliable and feasible than previous methods.

More importantly, the analysis that revealed comprehensive global frequency-spatial-temporal dependencies within the extensive EEG network demonstrated the interpretability of our proposed models, providing insights into how our models learn from voice-related EEG signals and how they relate to the neural processes underlying impaired auditory-vocal integration in PD. Upon visualizing the overall representation acquired by our models and their corresponding saliency maps during vocal pitch regulation, the most prominent discriminatory distributions were located in the left vPMC, STG, and Broca’s area. These regions have been well-established as integral to speech motor control^41–46^. This consistency suggests that our GSP-GCNs models capture distinct patterns of brain activity during vocal motor control between PD patients and healthy controls.

EEG microstate analysis has been proven valuable in the diagnosis of Alzheimer’s disease^47^ and schizophrenia^13^ by revealing the spatiotemporal dynamics of brain activity. In the present study, the microstate analysis of voice-related EEG signals identified five distinct microstates (MS1-MS5), with MS5 being of particular significance. Our observation of distinct MS5 microstate between PD patients and healthy controls is in line with previous studies that have shown associations of specific EEG microstates with vocal tract muscles and motor cortex activity^48,49^ as well as speech fluency in PD patients^50^. MS5 corresponds to the large-scale EEG networks that align with the P2 component (205–315 ms). This ERP component has been thought to reflect a complex stage of auditory-motor transformation for controlling vocal production that demands higher-level cognitive processing^51,52^. In particular, enhanced P2 responses to vocal pitch perturbations have been linked to impaired auditory-vocal integration in PD patients when compared to healthy controls^23–25^. Notably, the spatial localization of MS5 coincided with the left vPMC and Broca’s area (see Fig. 4), regions that have been recognized as significant contributors to enhanced P2 responses during vocal pitch regulation in PD patients compared to healthy controls^23^. More interestingly, this network representation of MS5 microstate also showed a resemblance to the discriminative distributions learned by the GSP-GCNs model, lending further support to the interpretability of our proposed models to unravel specific brain patterns during speech tasks relevant to PD. Therefore, this interpretability analysis highlights the novelty and significance of our approach in facilitating a more profound understanding of the neural mechanisms underlying PD. Such interpretability is crucial^27,28^, ensuring that the models are not just “black boxes” but provide meaningful insights into the neural dynamics underpinning PD.

The proposed GSP-GCNs models have important clinical implications for PD diagnosis. They offer the potential to improve the diagnostic accuracy by extracting interpretable features from large-scale voice-related EEG networks. They also contribute to reducing the subjective bias and variability across patients, thereby promoting a more objective and consistent assessment of PD. Moreover, they hold the promise to facilitate the treatment of speech impairment in PD by modulating activity of the brain regions observed in microstate analysis through the use of non-invasive brain stimulation techniques such as transcranial magnetic or electrical stimulation^25,53–55^. Therefore, our GSP-GCNs models not only represent an advancement in PD diagnosis but also may pave the way for effective treatment approaches.

Online detection of clinical diseases is a challenging task that requires high accuracy and low latency for brain signals. However, most existing EEG systems for PD diagnosis are based on offline analysis of resting-state data, which suffers from high noise and low stability in real-time EEG signals due to factors such as subject concentration and device inconsistencies. Previous studies have demonstrated the effectiveness and adaptability of GCN models in real-world scenarios across various domains, such as online recommender systems^56^, traffic flow prediction^57^, and online animal tracking^58^. The present study proposed a hybrid approach that combined the computing capabilities of offline systems with the real-time responsiveness of online systems based on the voice-related EEG signals. In this framework, the offline system constructed the large-scale EEG networks to extract the features of specific ERP components, while the online system applied the GSP-GCNs model to enable real-time evaluation. This strategy achieved a balance between high accuracy and low latency in classification, allowing us to detect unique patterns within task-related EEG signals for online PD diagnosis.

This claim is supported by a posteriori verification of the proposed models (see Supplementary Fig. 1), which compares the performance of different deep learning models based on the random selection of trials from a pool of 100 trials for a voting selection process. Remarkably, the post-validation performance of our models exhibits only slight declines (4.1–4.9%) when compared to the results presented in Table 1. In contrast, previous studies^59,60^ have reported that online detection systems based on the brain signals typically experience an approximate 10% reduction in accuracy compared to their offline counterparts. Moreover, the GSP-CNSs models outperform both conventional deep learning models and previous EEG-based online detection systems^59,60^ in stochastic posterior performance and computational complexity (see Table 1 and Fig. 2). Therefore, our proposed models have high potential for online detection scenarios due to their high accuracy, low computational complexity, and objective evaluation.

There are several limitations in the present study that warrant further investigation. First, our sample size was relatively small due to difficulties in obtaining task-related EEG data under controlled experimental conditions and specialized equipment requirements, which may limit the generalizability and robustness of our models. Second, our models performed the connectivity analysis of large-scale EEG networks at the electrode level, which cannot provide precise anatomical sources that generate the EEG signals. Incorporating source localization analysis for constructing EEG connectivity network is therefore needed in future studies to provide a more accurate representation of functional interactions within the brain. Also, the exclusive use of GCN models in the present study may not be suitable for all types of graph data. Future studies should consider alternative methodologies (e.g. graph attention networks, graph neural ordinary differential equations) for graph learning. Lastly, our models focused on a specific task that involves vocal motor control for PD diagnosis. Other tasks, such as cognitive control, may reveal different brain activity patterns and network dynamics that can be informative for PD diagnosis.

In conclusion, the present study proposed a novel deep learning framework based on GSP-GCNs for PD diagnosis using voice-related EEG signals. Our models can capture the global frequency-spatial-temporal dependencies among large-scale EEG networks, achieving a remarkable 90.2% classification accuracy and outperforming other deep learning models by 9.5%. Moreover, our models revealed the discriminative distributions of large-scale EEG networks and topography of microstate MS5 for PD diagnosis in terms of interpretability. These findings highlight the promise of interpretable deep learning models with task-related EEG signals in advancing PD diagnosis.

## Methods

### Task-related EEG Dataset

Fifty-two patients diagnosed with idiopathic PD (24 females and 28 males; mean age = 64.23 ± 5.30 years) according to the diagnostic criteria of the UK Parkinson’s disease Society Brain Bank^61^ and forty-eight sex- and age-matched healthy controls (HC) (23 females and 25 males; mean age = 63.37 ± 5.41 years) participated in this study. All of them were right-handed, native Mandarin speakers. PD patients in the present study met the following inclusion criteria: no more than mild dementia (Mini-Mental State Examination [MMSE] > 26), no other neurological disease, no history of neurosurgical treatment, laryngeal surgery or pathology, swallowing disorders. PD patients were kept on their antiparkinsonian medication, but they were tested during their off-medication state (i.e. 12 h off anti-PD medication). All participants provided informed consent, and the research protocol was approved by the Institutional Review Board of The First Affiliated Hospital at Sun Yat-sen University in accordance with the Code of Ethics of the World Medical Association (Declaration of Helsinki).

Task-related EEG data were acquired during a task based on the frequency altered feedback (FAF) paradigm^62^. In brief, participants were instructed to produce a sustained vowel sound (/u/) for a duration of 5–6 s while hearing their voice unexpectedly pitch-shifted downwards by 200 cents (100 cents = one semitone) for a duration of 200 ms. Each vocalization consisted of 4–5 perturbations that were presented in a pseudorandomized manner. Participants produced 20–25 consecutive vocalizations, resulting in a total of 100 trials. More details regarding experimental designs for the FAF tasks can be found in previous studies^23,25^.

While participants performed the vocal production experiment, the EEG signals were scalp-recorded using a 64-electrode Geodesic Sensor Net connected to a Net Amps 300 amplifier (Electrical Geodesics Inc.) at a sampling frequency of 1 kHz using NetStation software (v.4.5, Electrical Geodesics Inc.). During the offline analysis, the EEG signals were band-filtered (1–20 Hz) and segmented with a window of −200 ms before and 500 ms after the perturbation onset. An artifact detection procedure was applied to the segmented epochs to exclude those bad trials from further analysis. And artifact-free trials were re-referenced to the average of the electrodes on each mastoid, averaged, and baseline-corrected to generate an overall cortical ERP response.

### System framework

Figure 1 shows the overall framework of knowledge-guide graph convolutional networks, which consists of four modules: EEG network, GSP, GCN, and interpretable module. Firstly, the EEG network module constructs a graph of dynamic brain activity using electrodes as nodes and functional connections between electrodes as edges^63,64^. Subsequently, the GSP module applies a strategy based on incomplete network alignment (iNEAT)^65^ and Sparse Graph^66^ to reorganize the local and global information in the EEG network. Next, the GCNs module learns graph representations for personalized diagnosis by aggregating information from neighboring nodes, capturing intrinsic features from the complex EEG network for PD diagnosis. In addition, the interpretable module uses a modified saliency map derived from the backpropagation algorithm^67^ to visualize the prominent EEG network in each individual pattern and then compares it with the large-scale EEG network obtained from microstate analysis. The details of each module are described below.

#### EEG network definition

The EEG network is modeled by a graph ***G****(V,E)*, where *v*_*i*_ represents the *i*th channel of EEG in a node set *V* while *e*_*ij*_ represents the strength of functional connection between nodes *v*_*i*_ and *v*_*j*_ in an edge set *E*. Specifically, a single-channel EEG data records a sequence of time-series ***r***_***i****∈*[*1,n*]_=[***s***^*i*^_1_,***s***^*i*^_2_,…,***s***^*i*^_*k*_]*∈****R***^***k***^, where *k* = *T xf* denotes the number of time points (*f* represents the frequency range, and *T* represents the consecutive order of time series). The EEG signal can be represented as a tensor ***X***_***T***_ =[***r***_***1***_*;****r***_***2***_*;…;****r***_***n***_]*∈****R***^***nχk***^, and ${\boldsymbol{G}}$*(V,E)* can be represented as an adjacency matrix. Functional connectivity matrices (*V*) are calculated using Pearson Correlation Coefficient (PCC) or Phase Locking Value (PLV). The PCC between brain signals in different channels is defined by Eq. (1), where ${{\boldsymbol{r}}}_{{\boldsymbol{i}}}^{{{\boldsymbol{f}}}_{{\boldsymbol{p}}}}({\boldsymbol{t}})$ corresponds to the low energy fluctuation within the frequency band *f*_*p*_ (4–12 Hz) from the *i*th electrode of the EEG signals.

1 $${PCC}({{\boldsymbol{r}}}_{{\boldsymbol{i}}}^{{{\boldsymbol{f}}}_{{\boldsymbol{p}}}},{{\boldsymbol{r}}}_{{\boldsymbol{j}}}^{{{\boldsymbol{f}}}_{{\boldsymbol{q}}}})=\frac{1}{{N}_{s}}\mathop{\sum }\limits_{k=1}^{{N}_{s}}{{\boldsymbol{r}}}_{{\boldsymbol{i}}}^{{{\boldsymbol{f}}}_{{\boldsymbol{p}}}}({\boldsymbol{k}}){{\boldsymbol{r}}}_{{\boldsymbol{j}}}^{{{\boldsymbol{f}}}_{{\boldsymbol{q}}}}({\boldsymbol{k}})$$

PLV, defined in Eq. (2), measures the synchronization between phases of brain regions.

2 $${PLV}\left({{\boldsymbol{r}}}_{{\boldsymbol{i}}}^{{{\boldsymbol{f}}}_{{\boldsymbol{p}}}},{{\boldsymbol{r}}}_{{\boldsymbol{j}}}^{{{\boldsymbol{f}}}_{{\boldsymbol{q}}}}\right)=\left|\left\langle {e}^{{im}\Delta {{{\varnothing }}}_{{\boldsymbol{r}}}\left({\boldsymbol{t}}\right)}\right\rangle \right|=\left|\frac{1}{{N}_{s}}\mathop{\sum }\limits_{k=1}^{{N}_{s}}{e}^{{im}\left({{{\varnothing }}}_{{\boldsymbol{i}}}\left({\boldsymbol{k}}\right){\boldsymbol{-}}{{{\varnothing }}}_{{\boldsymbol{j}}}\left({\boldsymbol{k}}\right)\right)}\right|$$

$\Delta {\varnothing }_{{\boldsymbol{r}}}\left({\boldsymbol{t}}\right)$ is the instantaneous phase calculated by Hilbert transform from the original signal ${{\boldsymbol{r}}}^{{\boldsymbol{f}}}({\boldsymbol{t}})$.

The traditional GCNs focus on the local network structure rather than the global network distribution. To overcome this limitation, the present study incorporated a GSP method, including the iNEAT algorithm and Sparse Graph, into the GCNs. This integration allows us to capture both the local and global information within the EEG network. The iNEAT component selects edge features based on the hub and link properties of nodes, while the sparse graph component adjusts node weights according to edge weights. These methods enable a reorganization of the EEG network to obtain a new network that contains important nodes and edges while reducing or eliminating noisy ones. This reorganization is an important step in the GSP process for enhancing the data quality, as evidenced in previous studies on graph learning^68,69^. Although the iNEAT algorithm integrates both the local and global information within the large-scale EEG network, it may result in an issue of over completeness in fusion information. To overcome this limitation, the present study introduced the Sparse Graph operation, which performs sparse decomposition and dictionary generation on the graph signal, to remove redundant information.

Two graph networks, ***G***_***1***_ from a single-hop network (k = 1) and ***G***_***2***_ from a multi-hop network (k = 8), were generated for each individual using the k-nearest neighbor method^65^. The adjacency matrices ***A***_***1***_ and ***A***_***2***_ were calculated to obtain the permutation matrix S. To optimize the global graph information of the single-hop network, a graph matching-based method was proposed to solve a Non-deterministic Polynomial problem according to the principle of topology consistency^70,71^.

3 $$\mathop{\min }\limits_{{\boldsymbol{S}}}{{||}{{\boldsymbol{A}}}_{{\bf{2}}}-{{\boldsymbol{S}}}^{{\boldsymbol{T}}}{{\boldsymbol{A}}}_{{\bf{1}}}{\boldsymbol{S}}{||}}_{F}^{2}$$

where ${{||}\cdot {||}}_{F}$ is the Frobenius norm of the corresponding matrix.

The random walk-based method was used to capture collaborative graph information from both single- and multi-hop networks based on the Kronecker product graph^72,73^.

4 $${\boldsymbol{S}}={{\alpha }}\left({{\boldsymbol{A}}}_{{\bf{1}}}\otimes {{\boldsymbol{A}}}_{{\bf{2}}}\right){\boldsymbol{S}}+\left(1-\alpha \right){\boldsymbol{h}}$$

where ${\boldsymbol{h}}$ is the vectorization of the prior similarity matrix ***H*** via the sine function between mean adjacency matrices in different groups, and $\otimes$ is the operation of Kronecker product graph.

The iNEAT algorithm, which combines the strengths of the graph matching-based method and the random walk-based method^65^, was used in the present study. This algorithm effectively integrates the graph structure information from both single- and multi-hop networks within large-scale EEG networks. The optimization objective function is defined as follows:

5 $$\mathop{\min }\limits_{{\boldsymbol{s}}}\alpha\, {{\boldsymbol{s}}}^{T}\left({\boldsymbol{D}}-{{\boldsymbol{A}}}_{{\bf{1}}}\otimes {{\boldsymbol{A}}}_{{\bf{2}}}\right){\boldsymbol{s}}+(1-\alpha ){{||}{{\boldsymbol{D}}}^{\frac{{\bf{1}}}{{\bf{2}}}}\left({\boldsymbol{s}}-{\boldsymbol{h}}\right){||}}_{{\boldsymbol{F}}}^{{\bf{2}}}$$

where ${\boldsymbol{s}}$ represents the vectorization of the similarity matrix ***S***. ${\boldsymbol{D}}={{\boldsymbol{D}}}_{{\bf{1}}}\otimes {{\boldsymbol{D}}}_{{\bf{2}}}$ and ${{\boldsymbol{D}}}_{{\bf{1}}}$ and ${{\boldsymbol{D}}}_{{\bf{2}}}$ are the diagonal degree matrix corresponding to ${{\boldsymbol{A}}}_{{\bf{1}}}$, and ${{\boldsymbol{A}}}_{{\bf{2}}}$. In addition, a permutation matrix was used to reorganize the EEG signals through channel-wise operations at the individual level.

The Sparse Graph operation, defined in Eq. (6), aims to enhance the discriminative ability between groups and reduce the standard deviation across the trials. In Eq. (6), the parameter of ${{\boldsymbol{x}}}_{i}^{e}$ represents the EEG signal of *e*th electrode from the *i*th subject, while the graph matrix ${{\boldsymbol{w}}}_{i}^{e}$ is calculated through the iNEAT algorithm to learn the collaborative graph information.

6 $$\begin{array}{ll}{{\boldsymbol{W}}}^{e}={\arg}\mathop{\min }\limits_{{{\boldsymbol{W}}}^{e}}\mathop{\sum}\limits_{i=1}^{n}\left(\frac{1}{2}{{{||}}{{\boldsymbol{x}}}_{i}^{e}-{{\boldsymbol{X}}}_{i}^{e}{{\boldsymbol{w}}}_{i}^{e}{{||}}}_{2}^{2}\right)+{ \leftthreetimes}_{1}{{||}{{\boldsymbol{B}}}_{g}^{e}{\odot}{{\boldsymbol{W}}}^{{\boldsymbol{e}}}{||}}_{2,1}+{ \leftthreetimes }_{2}\mathop{\sum }\limits_{i,j=1}^{n}{s}_{{gij}}^{e}{{{||}}{{\boldsymbol{w}}}_{{gi}}^{e}-{{\boldsymbol{w}}}_{{gj}}^{e}{{||}}}_{2}^{2}\\\qquad+\,{ \leftthreetimes }_{3}{{{||}}{{\boldsymbol{B}}}_{t}^{e}{\odot}{{\boldsymbol{W}}}^{{\boldsymbol{e}}}{{||}}}_{2,1}+{ \leftthreetimes }_{4}\mathop{\sum }\limits_{i,j=1}^{n}{s}_{{tij}}^{e}{{{||}}{{\boldsymbol{w}}}_{{ti}}^{e}-{{\boldsymbol{w}}}_{{tj}}^{e}{{||}}}_{2}^{2}\end{array}$$

Where ${{\boldsymbol{B}}}_{g}^{e}=[{{\boldsymbol{b}}}_{g1}^{e},\ldots ,{{\boldsymbol{b}}}_{{gi}}^{e},\ldots ,{{\boldsymbol{b}}}_{{gn}}^{e}]$ is a weighting matrix with elements being ${{\boldsymbol{b}}}_{{gi}}^{e}=[{b}_{{gi}}^{e,1},\ldots ,{b}_{{gi}}^{e,e-1},{b}_{{gi}}^{e,e+1},\ldots ,{b}_{{gi}}^{e,E}]$. Similarly, the matrix ${{\boldsymbol{B}}}_{t}^{e}$ is defined. ${s}_{{gij}}^{e}$ and ${s}_{{tij}}^{e}$ denote the similarity between *i*th and *j*th subject from different groups.

#### Graph Convolutional Networks

In contrast to the spectrum-based approach based on the CNNs, the GCNs use the graph structure to intelligently aggregate information from neighboring nodes. Spectral analysis of graph signals decomposes EEG signals into multi-frequency graph modes and identifies the distribution of EEG signals using Laplacian maps from the spatial domain to the spectral domain. Laplacian operator, denoted as L, is defined as follows:

7 $${\bf{L}}={\bf{D}}-{\bf{A}}\in {{\mathbb{R}}}^{{\boldsymbol{N}}\times {\boldsymbol{N}}}$$

8 $${\bf{L}}={{\bf{I}}}_{{\boldsymbol{N}}}-{{\boldsymbol{D}}}^{-\frac{{\bf{1}}}{{\bf{2}}}}{\boldsymbol{A}}{{\boldsymbol{D}}}^{-\frac{{\bf{1}}}{{\bf{2}}}},{\boldsymbol{D}}\in {{\mathbb{R}}}^{{\boldsymbol{N}}\times {\boldsymbol{N}}}$$

where ${\boldsymbol{D}}$ is the diagonal degree matrix, and ${{\bf{I}}}_{{\boldsymbol{N}}}$ is an identity matrix of size N.

The graph signal can be defined through the Fourier transform^74^.

9 $${\boldsymbol{\chi }}={{\boldsymbol{U}}}^{{\boldsymbol{T}}}{\boldsymbol{x}},{\boldsymbol{x}}={\boldsymbol{U}}{\boldsymbol{\chi }}$$

where ${\boldsymbol{U}}=\left[{{\boldsymbol{u}}}_{{\bf{0}}},\ldots ,{{\boldsymbol{u}}}_{{\boldsymbol{N}}-{\bf{1}}}\right]\in {{\mathbb{R}}}^{{\boldsymbol{N}}\times {\boldsymbol{N}}}$ is calculated through the eigenvector decomposition of L.

In contrast to traditional methods that calculate a weighted sum of spatial neighbors in the Euclidean space, the ChebNet employed in the present study applied graph filters to generate a linear combination of graph Fourier modes across different frequencies. The convolution operation between two graph signals x and y can be expressed through the graph ${\mathscr{* }}{\mathcal{g}}$:

10 $${\boldsymbol{x}}* {\mathcal{g}}{\boldsymbol{y}}={\boldsymbol{U}}\Big(\left({{\boldsymbol{U}}}^{{\boldsymbol{T}}}{\boldsymbol{x}}\right)\odot\left({{\boldsymbol{U}}}^{{\boldsymbol{T}}}{\boldsymbol{y}}\right)\Big)$$

where ${\odot}$ represents the element-wise Hadamard product.

Since ${\bf{L}}={\boldsymbol{U}}{{\wedge }}{{\boldsymbol{U}}}^{{\boldsymbol{T}}}$ and ${{\wedge }}={\rm{diag}}\left(\left[{ \leftthreetimes }_{0},\ldots ,{ \leftthreetimes }_{N-1}\right]\right)$, we defined ${{\rm{g}}}_{\theta }$ as the filter with parameter θ. The filtering process of signal x can be expressed as:

11 $${\boldsymbol{y}}={{\rm{g}}}_{{\rm{\theta }}}\left({\bf{L}}\right){\boldsymbol{x}}={{\rm{g}}}_{{\rm{\theta }}}\left({\boldsymbol{U}}\wedge {{\boldsymbol{U}}}^{{\boldsymbol{T}}}\right){\boldsymbol{x}}={\boldsymbol{U}}{{\rm{g}}}_{{\rm{\theta }}}\left(\wedge \right){{\boldsymbol{U}}}^{{\boldsymbol{T}}}{\boldsymbol{x}}$$

To avoid calculating the spectral decomposition of the graph Laplacian, the ChebNet model used a truncated expansion of the Chebychev polynomials^74^.

12 $${{\rm{g}}}_{{\rm{\theta }}}\left(\wedge \right)=\mathop{\sum }\limits_{k=0}^{K-1}{\theta }_{k}{T}_{k}\left(\wedge \right)$$

13 $${\boldsymbol{y}}={\boldsymbol{U}}{{\rm{g}}}_{{\rm{\theta }}}\left(\wedge \right){{\boldsymbol{U}}}^{{\boldsymbol{T}}}{\boldsymbol{x}}=\mathop{\sum }\limits_{{\boldsymbol{k}}={\bf{0}}}^{{\boldsymbol{K}}-{\bf{1}}}{\theta }_{k}{\boldsymbol{U}}{T}_{k}\left(\wedge \right){{\boldsymbol{U}}}^{{\boldsymbol{T}}}{\boldsymbol{x}}=\mathop{\sum }\limits_{{\boldsymbol{k}}={\bf{0}}}^{{\boldsymbol{K}}-{\bf{1}}}{\theta }_{k}{T}_{k}\left({{\bf{L}}}^{\sim }\right){\boldsymbol{x}}$$

where ${{\bf{L}}}^{\sim }$ is scaled Laplacian: ${{\bf{L}}}^{\sim }={\bf{2}}{\bf{L}}/{ \leftthreetimes }_{{\boldsymbol{max }}}-{{\boldsymbol{I}}}_{{\boldsymbol{N}}}$.

#### Interpretable GSP-GCNs model

The present study used two methods to interpret the essential features derived from the GSP-GCNs model, including the examination of the features learned by the GSP-GCNs model and an analysis of the features of the large-scale EEG network through microstate analysis. The GSP-GCNs model was designed to capture global temporal dependencies within EEG data, enabling the identification of crucial information for decoding tasks from time series. While topography and Gradient-weighted Class Activation Mapping (CAM)^75^ have been used to reveal the global representation learned by deep learning models in motor imagery dataset^12^, the visualization of the gradients using the CAM often suffers from high levels of noise. To address this issue, the present study used a deconvolution approach that suppresses the flow of gradients through neurons^30^. Specifically, for a given layer *l* in the graph signal ${{\rm{\chi }}}^{l}$ and its gradient ${R}^{l}$, the overwritten gradient $\nabla {{\rm{\chi }}}^{l}{R}^{l}$ can be calculated as follows:

14 $$\nabla {{{\chi }}}^{l}{R}^{l}=\left({{{\chi }}}^{l} \,>\, 0\right)\odot\left(\nabla {{{\chi }}}^{l+1}{R}^{l+1} \,>\, 0\right)\odot\nabla {{{\chi }}}^{l+1}{R}^{l+1}$$

To generate the saliency map, the present study started from the output layer of a pre-trained model and propagated the gradients at each layer using the chain rule until reaching the input layer. This process allows us to visualize the salient regions that significantly contribute to the model’s predictions, providing valuable insights into the interpreted features.

The microstate analysis of ERPs aims to investigate whether the GSP-GCNs model can capture distinct neural representations between PD patients and healthy controls when they produce vocal compensations for pitch feedback perturbations. Microstate analysis is a method that assesses the functional dynamics of large-scale brain networks by identifying the stable topographic patterns of the EEG signals^47,50^. This analysis was performed using the MNE-python toolbox (https://mne.tools/stable/index.html), which involves calculating the global field power (GFP) of each participant’s ERP followed by clustering the topographic map underlying the GFP using the k-means algorithm from the *sklearn* toolbox. The Krzanowski-Lai criterion^76,77^ was used to determine the optimal number of microstate classes due to its suitability for selecting topographic map classifications based on quality indicators and global explained variance.

### Experimental design

The GSP-GCNs model was implemented using the Pytorch toolkit with a 5-fold cross-validation strategy (https://github.com/ShuzhiZhao/ERP_GCN). The model parameters were optimized using the Adam optimizer with gradient descent and the cross-entropy loss function. The network had three GCN layers (two hidden layers and one fully connected layer) and a learning rate of the network was 10^−5^. Table 2 shows the training parameters of the GCN model according to the Chebyshev polynomial order of each layer. A dropout rate of 0.35 was applied to prevent overfitting. The final output of the GSP-GCNs model was an *M*-dimensional vector obtained through the *Softmax* function, where *M* represents the number of EEG categories. The cross-entropy loss function, as defined in Eq. (15), was used to evaluate the model performance, where *y* and *yˆ* represent the ground truth and predicted label, respectively. *N*_*b*_ denotes the number of trials in a batch.

15 $${\mathcal{L}}{\mathscr{=}}{\mathscr{-}}\frac{1}{{N}_{b}}\mathop{\sum }\limits_{i=1}^{{N}_{b}}\mathop{\sum }\limits_{c=1}^{M}{ylog}\left({y}^{\wedge }\right)$$

**Table 2:** Number of training parameters of the GCN model according to the Chebyshev polynomial order of each layer.

Layers	Shape of weight tensor	Shape of bias	Number of parameters
Convolution layer_1_	[***K***_***1***_, 64, 64]	[64]	4096×***K***_***1***_ + 64
Pool_1_	[1×2]	[1×2]	4
Convolution layer_2_	[***K***_***2***_, 64, 32]	[32]	2048×***K***_***2***_ + 32
Pool_2_	[2×1]	[2×1]	4
Fully Connected layer	[32, 32]	[32]	1056

***K***_***1***_ and ***K***_***2***_ are the coefficients of the Chebyshev polynomial expansion.

To evaluate our proposed method, we compared it with several state-of-the-art approaches and performed ablation studies to show the impact of GSP. These baseline models used for comparison were Hybrid Convolutional Recurrent Neural Networks (CNN, RNN, 2D CNN-RNN, and 3D-CNN-RNN)^26^, EEGNet^34^, and CRNN (Cascade and Parallel model)^35^. The performance was evaluated using ACC, AUC, sensitivity, and specificity (1-specificity).

### Posteriori verification

A posteriori verification was performed to compare the classification performance of different deep learning models based on the random selection of trials from a pool of 100 trials for a voting selection process. The procedure consists the following steps:

Step 1: For each subject, 10 trials’ classification labels are randomly selected from a set of 100 trials’ classification labels ***Y*** = {*y*_*1*_, *y*_*2*_, *…*, *y*_*10*_}, where ${{\rm{y}}}_{i}\in \left\{0,1\right\}$ denotes the label of the *i*-th trial (0 represents PD patients, 1 represents healthy controls).

Step 2: A hard-voting strategy is used, where the total number of 0 labels ***N***_***0***_ and the total number of 1 labels ***N***_***1***_ in each subject’s 10 trials are computed. If ***N***_***0***_ > ***N***_***1***_, the subject is classified as having PD and the procedure progresses to Step 4. If ***N***_***0***_ < ***N***_***1***_, the subject is classified as healthy, bypassing to Step 4. If ***N***_***0***_ = ***N***_***1***_, the procedure advances to Step 3.

Step 3: The Boyer–Moore algorithm is applied for majority voting. For example, if ***Y*** = {*0*,*1*,*1*,*0,1,0,0*,*1*,*1*,*0*}, thus ***N***_***0***_ = ***N***_***1***_ occurs. The Boyer-Moore algorithm’s criterion is: the first trial vote is 0, the second trial vote is 1, the two votes are different and cancel each other; the second trial vote is 1, the third trial vote is 1, the two votes are the same, and the vote of ***BM***_***1***_ increases by 1. We get ***BM***_***0***_ = 1 and ***BM***_***1***_ = 2, and classify the subject as healthy.

Step 4: Steps 1–3 are reiterated 100 times, and the mean and variance of the outcomes from these repetitions are calculated to provide a statistical overview of the classification performance.

### Reporting summary

Further information on research design is available in the Nature Research Reporting Summary linked to this article.

### Supplementary information

Supplementary file

Reporting Summary

## Data Availability

Anonymized data may be shared on request to the corresponding author for non-commercial use, subject to restrictions according to participant consent and data protection legislation.

## Code availability

Code for all data analysis is available from the corresponding author upon request and establishment of data sharing agreement between institutions.
