# Supplementary material for: An interpretable model based on graph learning for diagnosis of Parkinson’s disease with voice-related EEG
Source: NPJ Digit Med. 2024 Jan 5;7:3. doi: 10.1038/s41746-023-00983-9 (PMC10770376; doi:10.1038/s41746-023-00983-9)

Supplementary Figure 1: The broken line chart that shows the stability assessment of a random posterior validation classifier based on Table 1. The classifier was trained using N trials that were randomly selected from the dataset, where N varied from 10 to 100. The training process was iterated 100 times for each N value. The vertical axis shows the evaluation metrics for the classifier (ACC, AUC, Sensitivity, and 1-Specificity), while the horizontal axis shows the number of randomly selected trials. Each colored line represents the performance of a specific classifier for different N values. The shaded area shows the variance of the performance across the 100 repetitions.

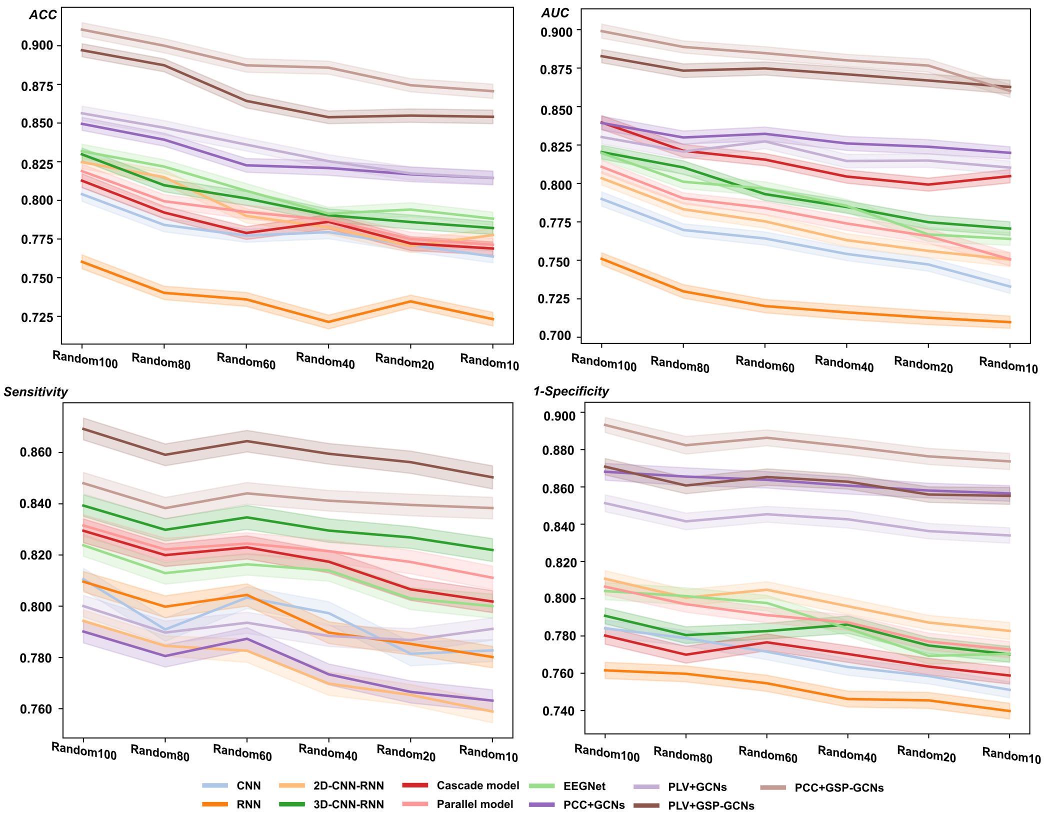

Supplement: Supplementary file 1 — Supplementary file [file 41746_2023_983_MOESM1_ESM.pdf]
