# Supplementary material for: An interpretable model based on graph learning for diagnosis of Parkinson’s disease with voice-related EEG
Source: NPJ Digit Med. 2024 Jan 5;7:3. doi: 10.1038/s41746-023-00983-9 (PMC10770376; doi:10.1038/s41746-023-00983-9)
Supplement: Supplementary file 2 — Reporting Summary [file 41746_2023_983_MOESM2_ESM.pdf]

nature portfolio

Corresponding author(s): Hanjun Liu

Last updated by author(s): Nov 15, 2023

Reporting Summary

Nature Portfolio wishes to improve the reproducibility of the work that we publish. This form provides structure for consistency and transparency in reporting. For further information on Nature Portfolio policies, see our Editorial Policies and the Editorial Policy Checklist.

Statistics

For all statistical analyses, confirm that the following items are present in the figure legend, table legend, main text, or Methods section.

n/a	Confirmed	
☐	☒	The exact sample size (*n*) for each experimental group/condition, given as a discrete number and unit of measurement
☐	☒	A statement on whether measurements were taken from distinct samples or whether the same sample was measured repeatedly
☐	☒	The statistical test(s) used AND whether they are one- or two-sided<br>*Only common tests should be described solely by name; describe more complex techniques in the Methods section.*
☒	☐	A description of all covariates tested
☒	☐	A description of any assumptions or corrections, such as tests of normality and adjustment for multiple comparisons
☒	☐	A full description of the statistical parameters including central tendency (e.g. means) or other basic estimates (e.g. regression coefficient) AND variation (e.g. standard deviation) or associated estimates of uncertainty (e.g. confidence intervals)
☐	☒	For null hypothesis testing, the test statistic (e.g. *F*, *t*, *r*) with confidence intervals, effect sizes, degrees of freedom and *P* value noted<br>*Give P values as exact values whenever suitable.*
☒	☐	For Bayesian analysis, information on the choice of priors and Markov chain Monte Carlo settings
☒	☐	For hierarchical and complex designs, identification of the appropriate level for tests and full reporting of outcomes
☒	☐	Estimates of effect sizes (e.g. Cohen's *d*, Pearson's *r*), indicating how they were calculated

Our web collection on statistics for biologists contains articles on many of the points above.

Software and code

Policy information about availability of computer code

Data collection	While participants performed the vocal production experiment, the EEG signals were scalp-recorded using a 64-electrode Geodesic Sensor Net connected to a Net Amps 300 amplifier (Electrical Geodesics Inc.) at a sampling frequency of 1 kHz using NetStation software (v.4.5, Electrical Geodesics Inc.).
Data analysis	The EEG data were analyzed using NetStation software (v.4.5) to extract ERPs to pitch perturbation in voice auditory feedback. The GSP-GCNs model was implemented using the Pytorch toolkit with a 5-fold cross-validation strategy (https://github.com/ShuzhiZhao/ERP_GCN).

For manuscripts utilizing custom algorithms or software that are central to the research but not yet described in published literature, software must be made available to editors and reviewers. We strongly encourage code deposition in a community repository (e.g. GitHub). See the Nature Portfolio guidelines for submitting code & software for further information.

Data

Policy information about availability of data

All manuscripts must include a data availability statement. This statement should provide the following information, where applicable:

- Accession codes, unique identifiers, or web links for publicly available datasets
- A description of any restrictions on data availability
- For clinical datasets or third party data, please ensure that the statement adheres to our policy

Provide your data availability statement here.

Research involving human participants, their data, or biological material

Policy information about studies with human participants or human data. See also policy information about sex, gender (identity/presentation), and sexual orientation and race, ethnicity and racism.

Reporting on sex and gender	Fifty-two patients diagnosed with idiopathic PD (24 females and 28 males) and forty-eight sex- and age-matched healthy controls (HC) (23 females and 25 males) participated in this study. The effects of sex on the ERPs to pitch perturbations were not investigated in the present study, since this is beyond the scope of the present study.
Reporting on race, ethnicity, or other socially relevant groupings	All of patients with PD and healthy controls were native Mandarin speakers from China.
Population characteristics	See above
Recruitment	Patients with PD were recruited from Department of Rehabilitation Medicine at The First Affiliated Hospital of Sun Yat-sen University, Guangzhou, China. Healthy controls were recruited from local communities near the hospital.
Ethics oversight	The research protocol was approved by the Institutional Review Board of The First Affiliated Hospital at Sun Yat-sen University, Guangzhou, China.

Note that full information on the approval of the study protocol must also be provided in the manuscript.

Field-specific reporting

Please select the one below that is the best fit for your research. If you are not sure, read the appropriate sections before making your selection.

☐ Life sciences ☒ Behavioural & social sciences ☐ Ecological, evolutionary & environmental sciences

For a reference copy of the document with all sections, see nature.com/documents/nr-reporting-summary-flat.pdf

Behavioural & social sciences study design

All studies must disclose on these points even when the disclosure is negative.

Study description	The present study proposes a novel and interpretable deep learning model, graph signal processing-graph convolutional networks (GSP-GCNs), using event-related EEG data obtained from a specific task involving vocal pitch regulation for Parkinson's disease diagnosis.
Research sample	Fifty-two patients diagnosed with idiopathic Parkinson's disease (PD) (24 females and 28 males; mean age=64.23) and forty-eight sex- and age-matched healthy controls (HC) (23 females and 25 males; mean age=63.37) participated in this study.
Sampling strategy	The sample size were determined based on previous studies that applied machine learning techniques for the diagnosis of Parkinson's disease using the task-related EEG signals.
Data collection	Participants were instructed to produce a sustained vowel sound (/u/) for a duration of 5-6 seconds while hearing their voice unexpectedly pitch-shifted downwards by 200 cents (100 cents = one semitone) for a duration of 200 ms. While participants performed the vocal production experiment, the EEG signals were scalp-recorded using a 64-electrode Geodesic Sensor Net connected to a Net Amps 300 amplifier (Electrical Geodesics Inc.) at a sampling frequency of 1 kHz using NetStation software (v.4.5, Electrical Geodesics Inc.).
Timing	Participants were instructed to produce a sustained vowel sound (/u/) for a duration of 5-6 seconds while hearing their voice unexpectedly pitch-shifted downwards by 200 cents for a duration of 200 ms. Each vocalization consisted of 4-5 perturbations that were presented in a pseudorandomized manner. Participants produced 20-25 consecutive vocalizations, resulting in a total of 100 trials.
Data exclusions	An artifact detection procedure was applied to the segmented epochs of EEG signals to exclude those bad trials from further analysis.
Non-participation	No participants dropped out the experiment and all of their EEG data were included for analysis in the present study.
Randomization	Participants were not allocated into experimental groups. And the data from PD patients and healthy controls were compared.

Reporting for specific materials, systems and methods

We require information from authors about some types of materials, experimental systems and methods used in many studies. Here, indicate whether each material, system or method listed is relevant to your study. If you are not sure if a list item applies to your research, read the appropriate section before selecting a response.

Materials & experimental systems

n/a	Involved in the study	
☒	☐	Antibodies
☒	☐	Eukaryotic cell lines
☒	☐	Palaeontology and archaeology
☒	☐	Animals and other organisms
☐	☒	Clinical data
☒	☐	Dual use research of concern
☒	☐	Plants

Methods

n/a	Involved in the study	
☒	☐	ChIP-seq
☒	☐	Flow cytometry
☒	☐	MRI-based neuroimaging

Clinical data

Policy information about clinical studies

All manuscripts should comply with the ICMJE guidelines for publication of clinical research and a completed CONSORT checklist must be included with all submissions.

Clinical trial registration	This is not a clinical trial study.
Study protocol	The research protocol was approved by the Institutional Review Board of The First Affiliated Hospital at Sun Yat-sen University in accordance with the Code of Ethics of the World Medical Association.
Data collection	Participants were instructed to produce a sustained vowel sound (/u/) for a duration of 5-6 seconds while hearing their voice unexpectedly pitch-shifted downwards by 200 cents (100 cents = one semitone) for a duration of 200 ms. While participants performed the vocal production experiment, the EEG signals were scalp-recorded using a 64-electrode Geodesic Sensor Net connected to a Net Amps 300 amplifier (Electrical Geodesics Inc.) at a sampling frequency of 1 kHz using NetStation software (v.4.5, Electrical Geodesics Inc.).
Outcomes	There is no expectation of primary or secondary treatment outcome, since this is not a clinical trial but a study for PD diagnosis using interpretable deep learning models.

Plants

Seed stocks	N/A
Novel plant genotypes	N/A
Authentication	N/A
